# Editorial: New advances in functional rehabilitation after central and peripheral nervous system injury

**DOI:** 10.3389/fneur.2023.1160382

**Published:** 2023-03-16

**Authors:** Ying Ding, Ge Li, Peixun Zhang, Wei Zhang

**Affiliations:** ^1^Department of Histology and Embryology, Zhongshan School of Medicine, Sun Yat-sen University, Guangzhou, China; ^2^Institute of Spinal Cord Injury, Sun Yat-sen University, Guangzhou, China; ^3^Key Laboratory for Stem Cells and Tissue Engineering, Ministry of Education, Sun Yat-sen University, Guangzhou, China; ^4^Advanced Medical Technology Center, The First Affiliated Hospital, Zhongshan School of Medicine, Sun Yat-sen University, Guangzhou, Guangdong, China; ^5^Guangdong Provincial Key Laboratory of Pathogenesis, Targeted Prevention and Treatment of Heart Disease, Guangdong Provincial People's Hospital (Guangdong Academy of Medical Sciences), Southern Medical University, Guangzhou, China; ^6^Key Laboratory of Trauma and Neural Regeneration, Ministry of Education, Department of Trauma and Orthopedics, Peking University People's Hospital, Peking University, Beijing, China; ^7^Research Department, Microbiome & Neuroscience, National Neuroscience Institute (NNI), Singapore, Singapore

**Keywords:** stroke, spinal cord injury, peripheral nerve injury, stem cell transplantation, biomaterials, electrical stimulation, acupuncture, neurogenesis

Nervous system injury, including central nerve injury and peripheral nerve injury, often leads to the loss of a patient's intelligence along with motor and sensory functions, and places a heavy burden on both the patient and their family. In adults, severe nerve injury can create significant difficulty for functional rehabilitation. The main reason for this is the death of neurons, demyelination, and nerve fiber regeneration failure at the site of injury. The nervous system is composed of neural tissue, which includes neurons, astrocytes, oligodendrocytes, microglia, and Schwann cells. The transmission of neural information between cells is achieved through complex crosstalk. At present, the regulation and remodeling mechanisms that each cell undergoes following injury to the nervous system remain very unclear. However, significant advances have been made in our understanding of functional rehabilitation following injury to the central and peripheral nervous systems (1). These new advances have reduced the possibility and intensity of side effects, and more importantly, have improved patients' quality of life.

Stroke is a complex disease that remains a global concern and results in nerve cell death and severe inflammation of the brain. According to the systematic review by Wang et al. included in this Research Topic, bone marrow mesenchymal stromal cells (BMSCs) provide an innovative strategy for stroke patients. In the central nervous system, transplanted BMSCs can promote M2 phenotype polarization of the microglia, resulting in the secretion of anti-inflammatory cytokines. Furthermore, the co-culture of BMSCs and astrocytes diminished the apoptosis of astrocytes and enhanced the level of neuroprotection. In the peripheral immune system, BMSCs and regulatory T cells (Treg) have been shown to provide immunomodulatory and neuroprotective functionality, respectively. From a molecular aspect, the transplantation of BMSCs to a lesion can result in the downregulation of several pro-inflammatory cytokines (IL-1, IL-6, TNF-α, and IFN-γ) and increased expression levels of anti-inflammatory cytokines, including IL-10 and TGF-β. Moreover, BMSCs may regulate neuroinflammation *via* growth factors or neurotrophins such as HGF and VEGF. Thus, BMSCs confer key neuroprotective effects after stroke.

Nerve regeneration is of vital importance when treating stoke. The activation of neurotrophic factors, along with their receptors and downstream pathways, is crucial (2). In this Research Topic, Mu et al. reviewed growth factors, such as nerve growth factor (NGF), brain-derived neurotrophic factor (BDNF), and glial cell lineage-derived neurotrophic factor (GDNF), that are involved in many molecular pathways and that promote the progression of neurogenesis after stroke (Figure 1). However, other factors, such as the myelin proteins Nogo-A, MAG, and OMgp, are thought to inhibit neurogenesis by disturbing the local microenvironment. In addition, the authors also reviewed the research progress on the use of acupuncture to improve functional recovery by promoting neurogenesis after stroke.

**Figure 1:**
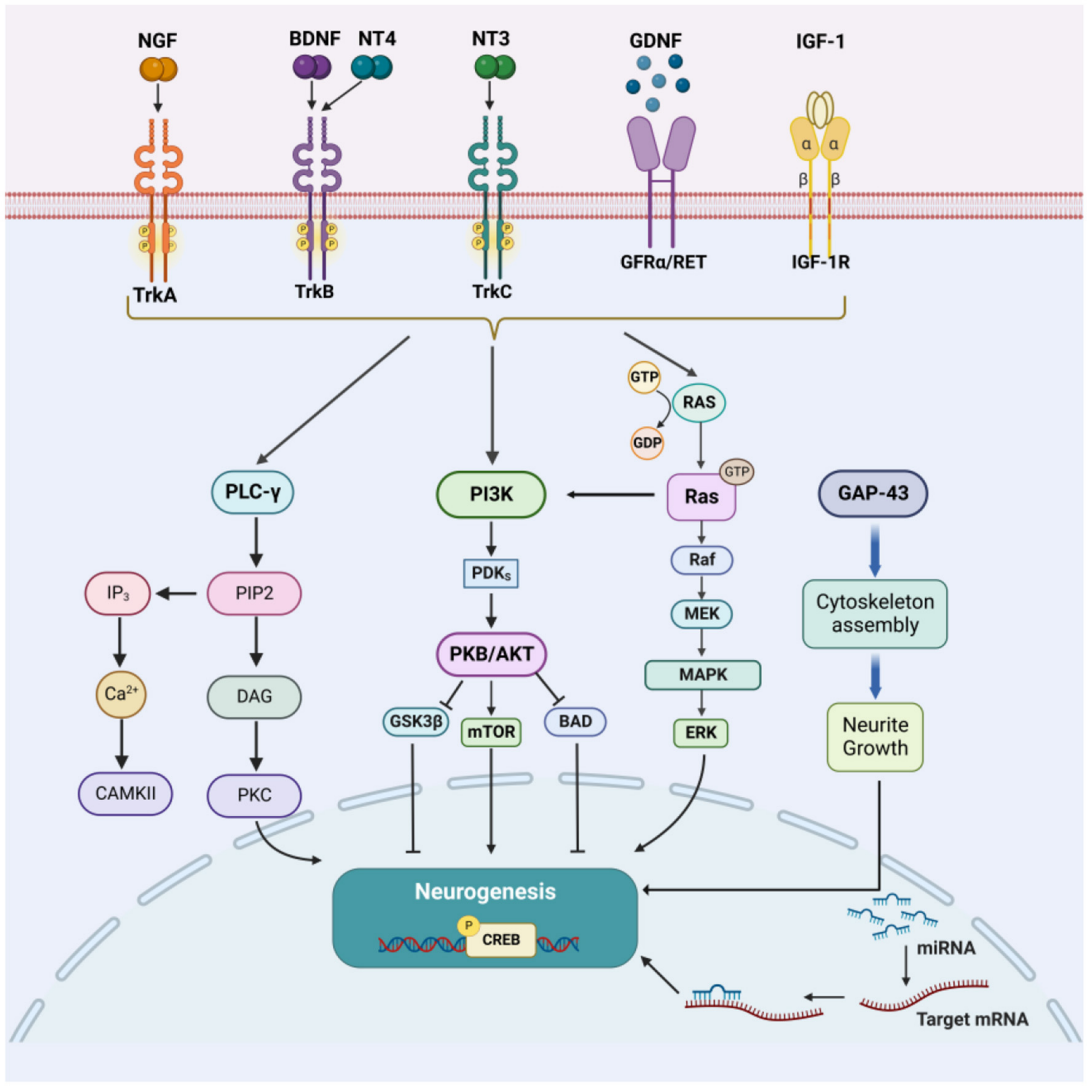
Diagram showing the factors that enhance neurogenesis and their major downstream signaling pathways following brain injury and stroke. Reprinted from Mu et al.

Apart from the direct injury site in the spinal cord, a series of secondary degenerative changes can spread across the entire neuroaxis from the spinal cord to the brain. Previously, researchers found that brain alterations following spinal cord injury (SCI) are associated with lesion severity and long-term outcomes. Thus, Yu et al. conducted a voxel-based meta-analysis to analyze a large dataset. The analysis showed that SCI patients exhibited a significant loss of gray matter volume (GMV) in the left insula and bilateral thalamus, along with a significant loss of white matter volume (WMV) in the bilateral corticospinal tract (CST). Their study was the first voxel-based meta-analysis to use SDM-PSI to evaluate morphological changes in the brains of patients after SCI. This technique estimates the severity of SCI in a visual manner and promotes the development of personalized treatment and clinical management.

As this technique matures, the treatment of nerve disease may become less sophisticated. Yu et al. reported advances in microvascular decompression (MVD) for classical trigeminal neuralgia (CTN) in terms of imaging and endoscopic techniques. High-resolution contrast-enhanced magnetic resonance imaging (MRI) can reveal and evaluate the location, degree, and range of neurovascular compression (NVC). Once the significantly responsible vessel has been identified by imaging, MVD can be targeted by a neurosurgeon. Furthermore, endoscopy provides a more optimized visualization of MVD as well as allowing a shorter surgical incision, thus reducing the possibility of postoperative headache, hearing loss, and other complications. Using this technique, possible pathogenic NVC vessels can be detected more reliably.

Peripheral nerve injuries can be caused by traffic accidents, sports, or other violent events. Trauma leads to nerve rupture, which often requires autologous nerve transplantation to reconstruct continuous nerve circuits. However, autologous nerve transplantation is still limited by a number of key hurdles, including nerve unavailability, size mismatch, and local tissue adhesion. To overcome these problems, Ye et al. developed a potential substitute material, which is referred to as decellularized tissue. This tissue substitute can provide a platform for cell adhesion, and its effects on tissue repair and the coordination of growth factors, cytokines, and chemokines, have already been proven. Of these decellularized tissues, decellularized nerve tissue (dN) is considered superior due to its homology; however, it is limited by longer peripheral nerve deficits. Thus, the authors selected decellularized kidney tissue (dK), which has been rarely studied, and compared this with dN. The authors found that dK played a more significant role in promoting axonal growth and the extension of neural-like cells, thus indicating that dK represents a promising bio-scaffold for nerve regeneration.

Acupuncture, as a traditional form of Chinese medicine, now plays an unexpected role in the treatment of nerve diseases. By inserting needles into the skin or deep tissues at specific locations (acupoints) on the body, the nerve endings beneath can be stimulated. A recent study showed that electroacupuncture treatment can regulate the expression of 173 mRNA, 260 lncRNA, and 153 circRNA differentially expressed genes after SCI, which are highly related to the signal pathways of inflammation, oxidative stress, apoptosis, and neuroprotection (3). In this Research Topic, Feng et al. conducted a meta-analysis to evaluate the effects of acupuncture on pain intensity and safety in patients suffering from neuropathic pain (NP). The analysis showed that the pain relief experienced by the acupuncture group was better than the sham intervention group or blank control group, although there was no significant difference between acupuncture and conventional drug treatments with regards to the treatment of NP. Moreover, acupuncture-induced adverse events were mild and reversible when compared to drug therapy, thus indicating that acupuncture is a relatively safe intervention for patients.

Finally, in recent years, crosstalk between the nerve system and bone has drawn the attention of researchers from different specialties (4, 5). For example, Liu et al. systematically reviewed the structural and functional interrelationships between the nerve system and bone. Innovatively, the authors developed a three-dimensional hard tissue clearing based on the polyethylene glycol (PEG)-associated solvent system, which can provide a more straightforward view of the nerve distribution in bone. Although we have a good understanding of how bone-related diseases can trigger pain, further research is needed to establish the specific mechanisms involved. Furthermore, postoperative nerve recovery and pain relief should be considered when it comes to treatment strategy. Liu et al. concluded that nerve fibers, growth factors, and neural-related cells are indispensable in the process of bone regeneration. Thus, we are more likely to witness a significant breakthrough in the repair of bone defects *via* nerve-related mechanisms.

Thanks to the efforts made by the abovementioned researchers, key mysteries in the field of nerve regeneration are finally being unraveled. The collection of studies presented in this Research Topic relating to the treatment of central and peripheral nervous diseases has provided some key new strategies and techniques.

## Author contributions

YD and GL prepared the original draft. PZ and WZ critically reviewed and edited the manuscript. All authors have reviewed and approved the final manuscript.
